# AGING IN PLACE IN BRAZIL: SOME NEW EMPIRICAL EVIDENCE AND REMARKS ON THE ROLE OF ENVIRONMENTS FOR EDUCATION

**DOI:** 10.1093/geroni/igae098.1280

**Published:** 2024-12-31

**Authors:** Frank Oswald, Johannes Doll, Lycia Tramujas Vasconcellos Neumann

**Affiliations:** Chair; Co-Chair; Discussant

## Abstract

Aging in Place and the changing role of place for aging in modern societies has become an increasingly important topic both from an empirical and applied perspective. Studies have demonstrated effects of improving the environment, however, research on complex interactions of place, aging and education is scarce, although particularly a tradition of educational theory exists in Brazil. This symposium aims to address links between place, aging, and education in later life in Brazil for health and well-being. The first contribution by Frank Oswald aims to rethink place and aging relationships and to focus on enduring and novel challenges in this regard to provide an overview over selected theories in environmental gerontology and beyond. Second, Mariana Alves da Silva do Nascimento presents qualitative data from a study conducted in São Paulo with community residents on perceptions, preferences, and expectations of aging in place from an Architecture and Urban Planning perspective. The third contribution by Sérgio Luiz Valente Tomasini uses a participatory approach to shed light on planning of outdoor spaces (gardens) within Long-Term Care Facilities in Brazil, emphasizing that participatory methods can positively impact adaptive outcomes of older adults in institutional settings. Fourth, Johannes Doll and Estela Kohlrausch emphasize the significance of educational aspects for environmental gerontological perspectives on place and aging in societies that face globalization, urbanization, digitalization and climate change, which can help to better understand environment in a broader context by “reading the world” (Paulo Freire) differently. Finally, Lycia Tramujas Vasconcellos Neumann will serve as the session’s discussant.

